# Using Z-score to optimize population-specific DDH screening: a retrospective study in Hangzhou, China

**DOI:** 10.1186/s12891-021-04216-6

**Published:** 2021-04-12

**Authors:** Haomin Li, Liqi Shu, Jin Yu, Zeng Xian, Huilong Duan, Qiang Shu, Jingjing Ye

**Affiliations:** 1 Clinical Data Center, National Clinical Research Center for Child Health, The Children’s Hospital, Zhejiang University School of Medicine, Binsheng Road 3333#, Hangzhou, 310052 China; 2 Rhode Island Hospital, Brown University, Providence, USA; 3 Department of Ultrasound, The Children’s Hospital, Zhejiang University School of Medicine, Binsheng Road 3333#, Hangzhou, 310052 China; 4 The College of Biomedical Engineering and Instrument Science, Zhejiang University, Hangzhou, China

**Keywords:** DDH screening, Graf typing, Z-score, False positive

## Abstract

**Background:**

DDH (Developmental Dysplasia of the Hip) screening can potentially avert many morbidities and reduce costs. The debate about universal vs. selective DDH ultrasonography screening in different countries revolves to a large extent around effectiveness, cost, and the possibility of overdiagnosis and overtreatment. In this study, we proposed and evaluated a Z-score enhanced Graf method to optimize population-specific DDH screening.

**Methods:**

A total of 39,710 history ultrasonography hip examinations were collected to establish a sex, side specific and age-based Z-scores model using the local regression method. The correlation between Z-scores and classic Graf types was analyzed. Four thousand two hundred twenty-nine cases with follow-up ultrasonographic examinations and 5284 cases with follow-up X-ray examinations were used to evaluate the false positive rate of the first examination based on the subsequent examinations. The results using classic Graf types and the Z-score enhanced types were compared.

**Results:**

The Z-score enhanced Graf types were highly correlated with the classic Graf’s classification (R = 0.67, *p* < 0.001). Using the Z-scores ≥2 as a threshold could reduce by 86.56 and 80.44% the false positives in the left and right hips based on the follow-up ultrasonographic examinations, and reduce by 78.99% false-positive cases based on the follow-up X-ray examinations, respectively.

**Conclusions:**

Using an age, sex and side specific Z-scores enhanced Graf’s method can better control the false positive rate in DDH screening among different populations.

**Supplementary Information:**

The online version contains supplementary material available at 10.1186/s12891-021-04216-6.

## Background

DDH (Developmental Dysplasia of the Hip) is a common pediatric orthopedic condition [1]. It represents a broad spectrum of conditions, ranging from congenital dislocation of the hips to occult acetabular dysplasia [2]. Left untreated, DDH can lead to long-term morbidities, including chronic pain, gait abnormalities, and degenerative arthritis. In patients with early diagnosis, within 3 to 6 months of life, the treatment which is essentially conservative and involves the use of dynamic harness, was reported with good clinical and ultrasonographic outcomes [3, 4]. As early and accurate diagnosis of DDH is believed to be the most important factor for satisfactory treatment, hip US (ultrasonography) has become the most commonly used diagnostic tool for DDH during early infancy and has been so for many years [5, 6]. The most widely used DDH screening method was developed by Reinhard Graf in the early 1980s [7–9]. The Graf classifications were based on several thresholds of the angles of α and β, as summarized in Supplemental Table S1 (see Additional file 1).

However, there is still controversy concerning the methodology used in infant hip screening programs such as the optimal screening time and the accuracy of the Graf classifications [10–12]. The results of an ultrasonographic study revealed that, among the Graf type IIa or worse hips that were identified during the first 3 days of life, only 9% would remain abnormal and require treatment during the follow-up period [13]. In the selective sonographic assessment of ‘at risk hips’ at 6 weeks, there was still a significant risk of overdiagnosis and over-treatment with a positive predictive value of 20.5% [10]. The high false-positive rate is also the major concern about universal DDH screening in many countries when considering costs and efficiency. There are several reasons for the high false-positive rate of the Graf method in early DDH screening. First, the thresholds of angles of α and β ignore the significant differences of race, age, sex, and sides of the hips. In addition to the rapid development of the hip during the first 3 months of infancy, there are notable differences between boys and girls and left and right hips. Furthermore, the measure differences of the angles of α and β among intraobserver and interobserver are of concern [14–18]. Thus the static thresholds for Graf typing, making the reported Graf types range from moderate to substantial and from fair to substantial, respectively [14–16, 19, 20]. In the following period, the Graf method together with the technology of the US machines improved dramatically. Other types of IIa Hips (IIa+,IIa-) have been introduced distinguishing immature hip and suspect pathologies in the first 3 months of life reducing the number of overtreatment. Some checklists were introduced to improve the reproducibility among intraobserver and interobserver [21, 22]. However, the literature surrounding the question about selective vs. universal US screening is still very varied around effectiveness, cost, and the possibility of overdiagnosis and overtreatment [23, 24]. A Cochrane review in 2013 concluded that neither US strategy had been demonstrated to improve clinical outcomes, including late diagnosed DDH and surgery [25]. But there also an international interdisciplinary consensus was published in 2019 that strong agreement in favor of universal US screening [26]. It seems different countries have different views on this issue and the debate has not ceased by far.

Based on many studies, there are different hip characteristics among races [27], between boys and girls [28], and the left side of hips are more commonly affected [29]. We also know the hip changes rapidly in the first 3 months after birth. But currently, the Graf method which is based on several static thresholds for all infants does not fully consider the difference of race, gender, age, and side of the hip.

Z-scores express how many SD (Standard Deviation) above (positive values) or below (negative values) a given measurement lies with respect to the mean of the specific population. A dynamic reference range based on the Z-score has been widely used in many clinical measurements, especially for fetuses and infants [34]. In pediatric practice, there is the added dimension of somatic growth: a single reference range cannot be applied across children of different races, sizes, sex, and age. For these reasons, we wanted to test whether an age, sex, and side specific Z-score enhanced Graf method could control the high false-positive rate in DDH screening.

## Methods

**Fig. 1:**
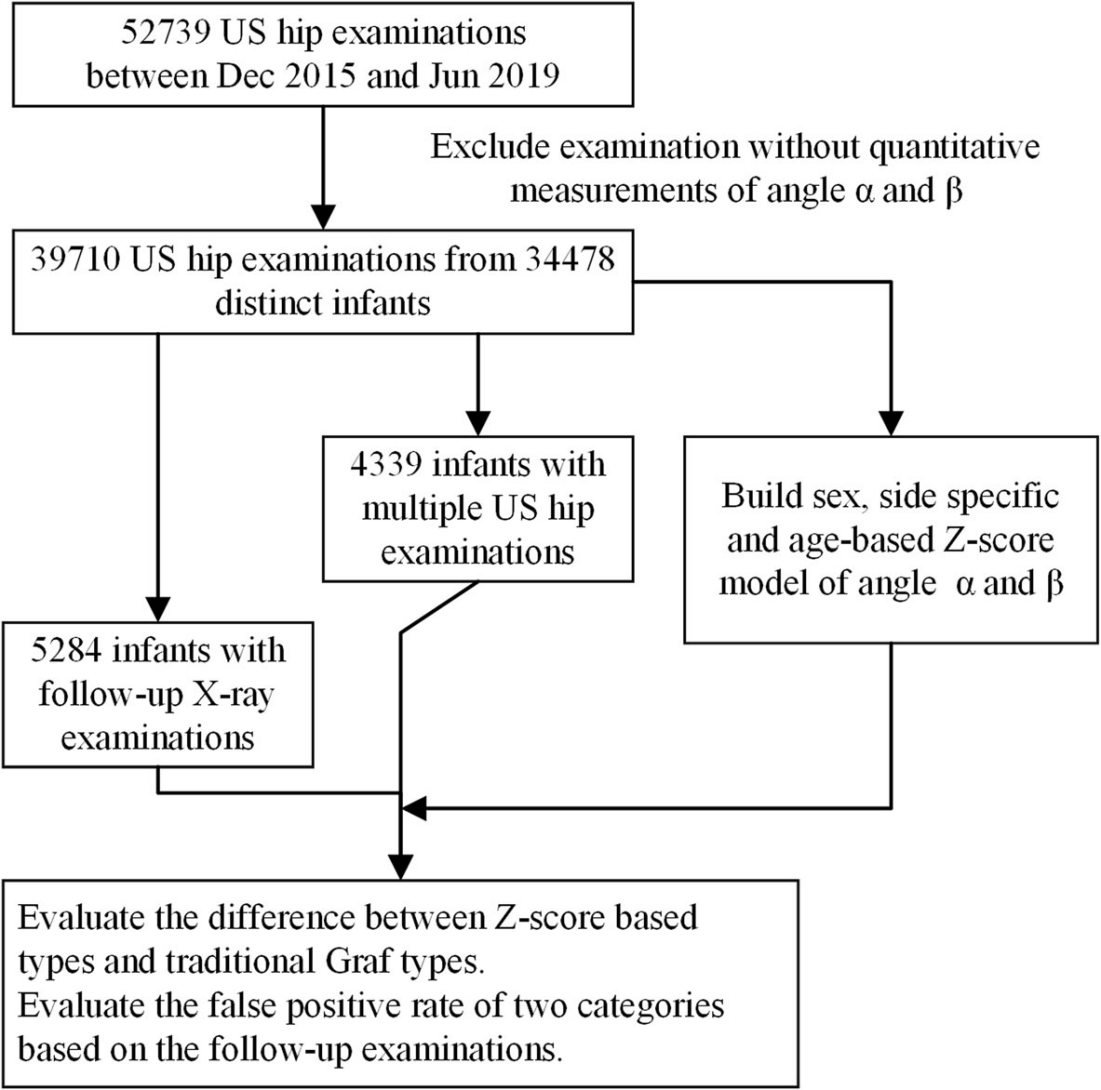
The patients and methods of this study

This retrospective observational study was approved by the Institutional Review Board/Ethics Committee of the Children’s Hospital of Zhejiang University School of Medicine. All research was performed in accordance with relevant guidelines/regulations. Written informed consent was waived by the Institutional Review Board/Ethics Committee, as utilization of anonymized retrospective data does not require patient consent under the local legislation. The patients and methods were summarized in Fig. 1.

The criteria of inclusion were patients with ultrasonographic hip examinations reports between Dec 2015 and Jun 2019 in the Children’s hospital. Some reports without quantitative measurements recorded based on Graf method were excluded. As the universal US screening program is not practiced in China, most of the infants who take the US hip examinations are suspected cases or risk cases. Clinical instability, clicky hip, asymmetric skin, breech presentation, family history, or other clinical problems such as foot deformity (including metatarsus adductus) were the main reason for this examination. All radiologists of these hip US examinations and X-ray examinations were trained and certified. The diagnosis criteria were following the clinical guidelines of DDH in China. A total of 39,710 reports from 34,478 distinct infants (45.49% male; mean age at examination 107.76 ± 169.36 days) were included in this study. The Graf types and α and β angles of two hips were extracted from the text report using a computer program that was developed locally. The extracted results were reviewed manually by the authors.

The datasets were split into 4 subsets based on sex and hip sides. For each dataset, the mean value and standard deviation (SD) of α and β for each age in days ranged from 1 to 200 days were calculated. A local regression (LOESS, R v3.4.0) approach was used to optimize the reference value and variance across the age range. Four Z-score models, for boys and girls, and left and right hips were generated. A Z-score calculator based on these models was developed using node.js and can be freely accessed online [31].

Based on these models, the Z-scores of the α and β angles from each examination were calculated for each hip separately. As we were only concerned about an α below the normal reference value in this study, the Z-scores of hips were rounded up and given as 0 to 5 Z-levels. Thus, Z-levels = 2 means the value is 2 SD below the mean value of the specific population, which also indicates that about 2.28% of infants in the population have a worse value in theory. The *cor.test* in R was used to test the Pearson’s correlation between the Z-levels of the α angle and the classic Graf types. The Cohen’s kappa value was used to measure the agreement of positive and negative between two categories.

As there are no gold standards for the DDH examinations, we defined two references based on follow-up examinations in this study. In the first reference, we used the follow-up ultrasonographic examination results as a gold standard to evaluate the false-positive rate of the first examination. In the studied population, there are 4229 infants (32.61% male, age at first examination 80.60 ± 75.01 days, age at last examination 135.84 ± 76.57 days, time interval 55.16 ± 28.94 days) with multiple ultrasonographic hip examinations. The Graf type IIa or worse hips were considered as positive results. A false-positive was defined as positive at the first examination but was negative at the follow-up examinations. In the second reference, we used the follow-up X-ray hip examination results as a gold standard. There are 5284 infants (39.88% male, age at ultrasonographic examination 107.30 ± 47.60 days, age at X-ray examination 261.50 ± 126.80 days, time interval 154.14 ± 126.07 days) with follow-up X-ray examinations. As the X-ray examinations do not measure the α and β angles, the X-ray reports with a clear statement of “no abnormality is revealed” will be considered as negative. The false-positive cases are cases which have a positive result in ultrasonographic examination but a negative result in X-ray examinations.

## Results

**Fig. 2:**
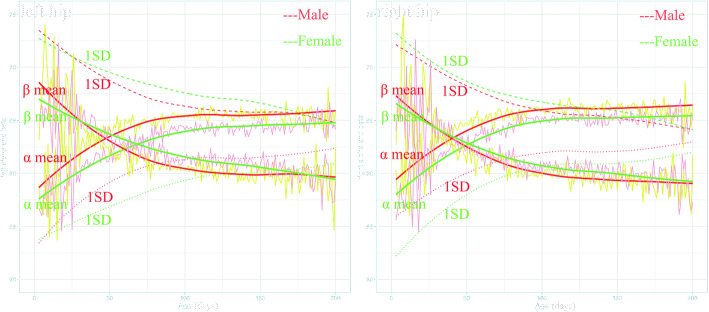
Z-score models of the α and β angles. The smoothed solid line is the modeled mean value (The polylines under it are the real mean value in this age group). The smoothed dotted and dashed lines represent the − 1 SD (standard deviation) of the angle α and + 1 SD of the angle β

As shown in Fig. 2, the mean values and corresponding 1 SD below the mean values of the α and 1SD above the mean values of β angles in the first 200 days of age based on the four Z-scores models for male and female and left and right hips were plotted. The mean value of α and β angles changed rapidly before the age of 100 days in all four models. The α and β angles were negatively correlated (Pearson’s R = − 0.13 in the left hip, *p* < 0.001; Pearson’s R = − 0.23 in the right hip, *p* < 0.001) in the 39,710 examinations. At the same time, it can be noted that there was a significant difference between boys and girls. The mean of the α angle in girls was smaller than that of the boys of all ages. The mean α angle of the right hip was larger than that of the left hip in both boys and girls of all ages (shown in Supplemental Fig. S1 (see Additional file 1)).

**Fig. 3:**
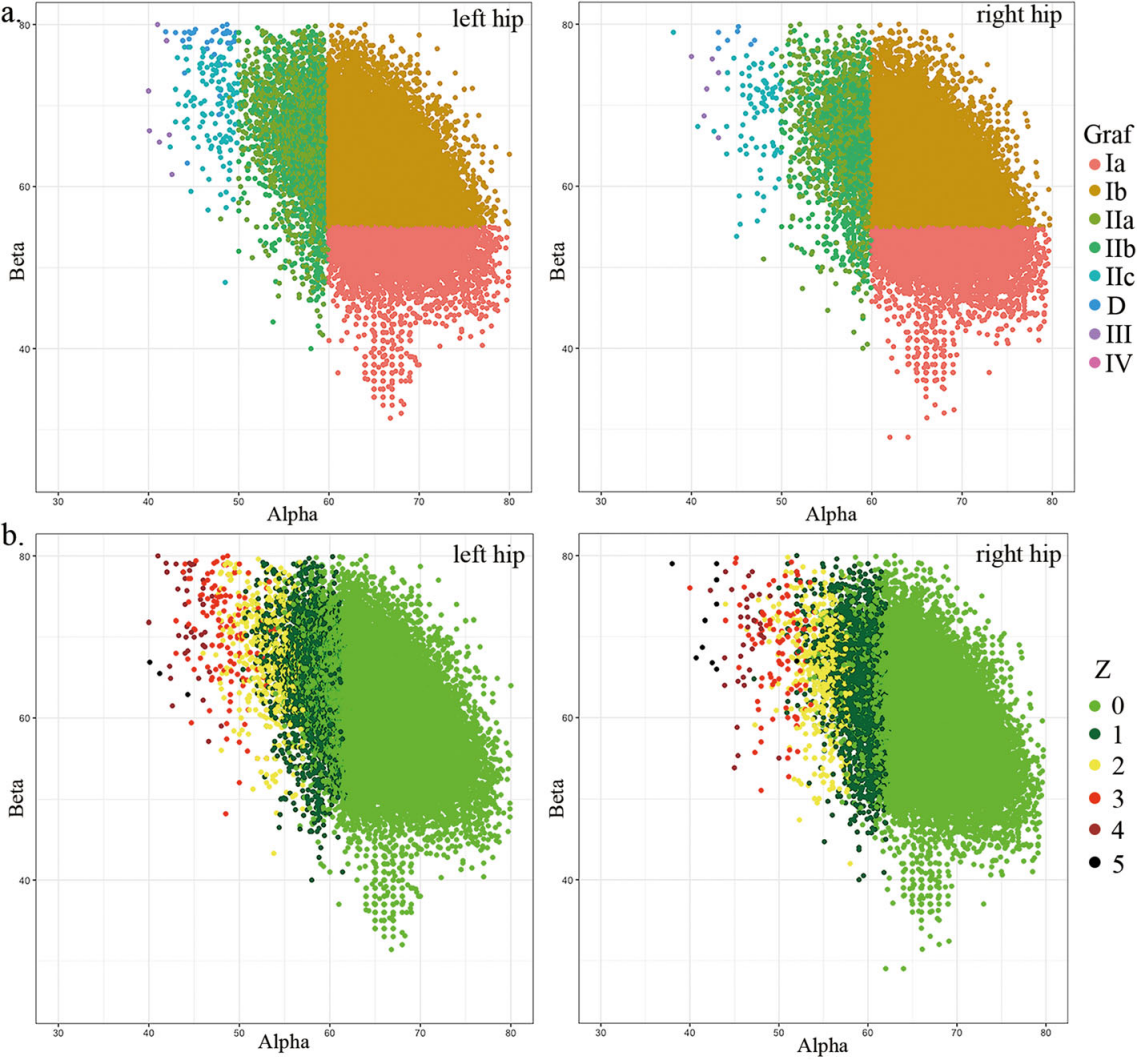
The α and β angles distribution of 34,478 infants US hip examinations. **a** Colored by Graf types with strict thresholds. **b** Colored by Z-levels that specific for age, sex, and side

The classic Graf types and Z-levels of the first ultrasonographic report of 34,478 infants are shown in Table 1 and Fig. 3. The Z-levels were highly correlated with the Graf types (Pearson’s R = 0.67 *p* < 0.001) as shown in Table 2 and the Z-levels with more flexible borders for different age boys and girls as shown in Fig. 3. Using Z ≥ 2 as the threshold, the Cohen’s kappa (k) = 0.271 and 0.374 in left and right hip respectively, which represents a fair strength of agreement between the two categories.

**Table 1:** The Graf type and Z-score results of first US examination of 34,478 infants

		Left Hips				Right Hips			
		Case #	Age (days)	Sex		Case #	Age (days)	Sex	
				Male	Female			Male	Female
Total		34,478	107.76 (±169.36)	15,683	18,795	34,478	107.76 (±169.36)	15,683	18,795
Graf	Ia	5130 (14.88%)	117.49 (±218.81)	2615 (16.67%)	2515 (13.38%)	5272 (15.29%)	119.72 (±240.33)	2592 (16.53%)	2680 (14.26%)
	Ib	25,549 (74.10%)	108.52 (±160.14)	12,045 (76.80%)	13,504 (71.85%)	26,748 (77.58%)	107.27 (±154.80)	12,424 (79.22%)	14,324 (76.21%)
	IIa	1868 (5.42%)	62.83 (±185.36)	518 (3.30%)	1350 (7.18%)	1273 (3.69%)	60.69 (±164.57)	353 (2.25%)	920 (4.89%)
	IIb	1717 (4.98%)	119.94 (±105.41)	475 (3.03%)	1242 (6.61%)	1079 (3.13%)	120.40 (±68.57)	299 (1.91%)	780 (4.15%)
	IIc	91 (0.26%)	79.15 (±39.29)	10 (0.06%)	81 (0.43%)	53 (0.15%)	76.00 (±34.42)	4 (0.03%)	49 (0.26%)
	D	50 (0.15%)	76.84 (±37.83)	3 (0.02%)	47 (0.25%)	19 (0.06%)	69.74 (±42.12)	3 (0.02%)	16 (0.09%)
	III	53 (0.15%)	83.23 (±40.43)	8 (0.05%)	45 (0.24%)	20 (0.06%)	65.05 (±33.71)	4 (0.03%)	16 (0.09%)
	IV	20 (0.06%)	77.00 (±42.89)	9 (0.06%)	11 (0.06%)	14 (0.04%)	91.57 (±35.75)	4 (0.03%)	10 (0.05%)
Z-level	0	30,479 (88.40%)	107.11 (±169.72)	13,748 (87.66%)	16,731 (89.02%)	30,245 (87.72%)	106.84 (172.33)	13,611 (86.79%)	16,634 (87.66%)
	1	3316 (9.62%)	111.97 (±152.97)	1692 (10.79%)	1624 (8.64%)	3622 (10.51%)	114.15 (139.50)	1854 (11.82%)	1768 (9.32%)
	2	485 (1.41%)	122.86 (±254.37)	195 (1.24%)	290 (15.43%)	478 (1.39%)	118.23 (195.94)	182 (1.16%)	296 (1.56%))
	3	99 (0.29%)	111.16 (±134.26)	24 (0.15%)	75 (0.40%)	72 (0.21%)	120 (155.14)	19 (0.12%)	53 (0.28%)
	4	47 (0.14%)	87.72 (±38.99)	9 (0.06%)	38 (0.20%)	32 (0.09%)	86.44 (37.35)	7 (0.04%)	25 (1.32%)
	5	20 (0.06%)	107.3 (±44.96)	5 (0.03%)	15 (0.08%)	12 (0.03%)	108.5 (31.65)	4 (0.03%)	8 (0.04%)
	NA	32 (0.09%)	84.22 (±46.03)	10 (0.06%)	22 (0.12%)	17 (0.05%)	79.59 (34.89)	6 (0.04%)	11 (0.06%)

**Table 2:**
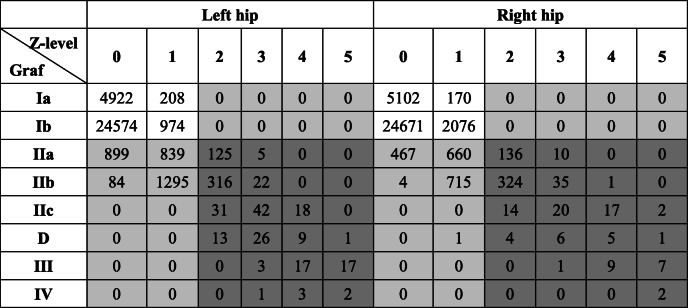
The correlation between Graf type and Z-level in 39,710 US examinations. The grey background color shows the positive result in two categories

In the 39,710 examinations, based on the classic Graf type, the positive rate was 14.24% (4910/34478) [left hip 11.02%; right hip 7.13%]. Based on the Z-scores (Z ≥ 2, which is widely accepted as a normal reference for many medical measurements), the positive rate was 3.33% (1147/34478) [left hip 1.98%; right hip 1.77%]. If using Z ≥ 1, the positive rate was 11.52 and 12.23% for the left and right hips, respectively.

**Fig. 4:**
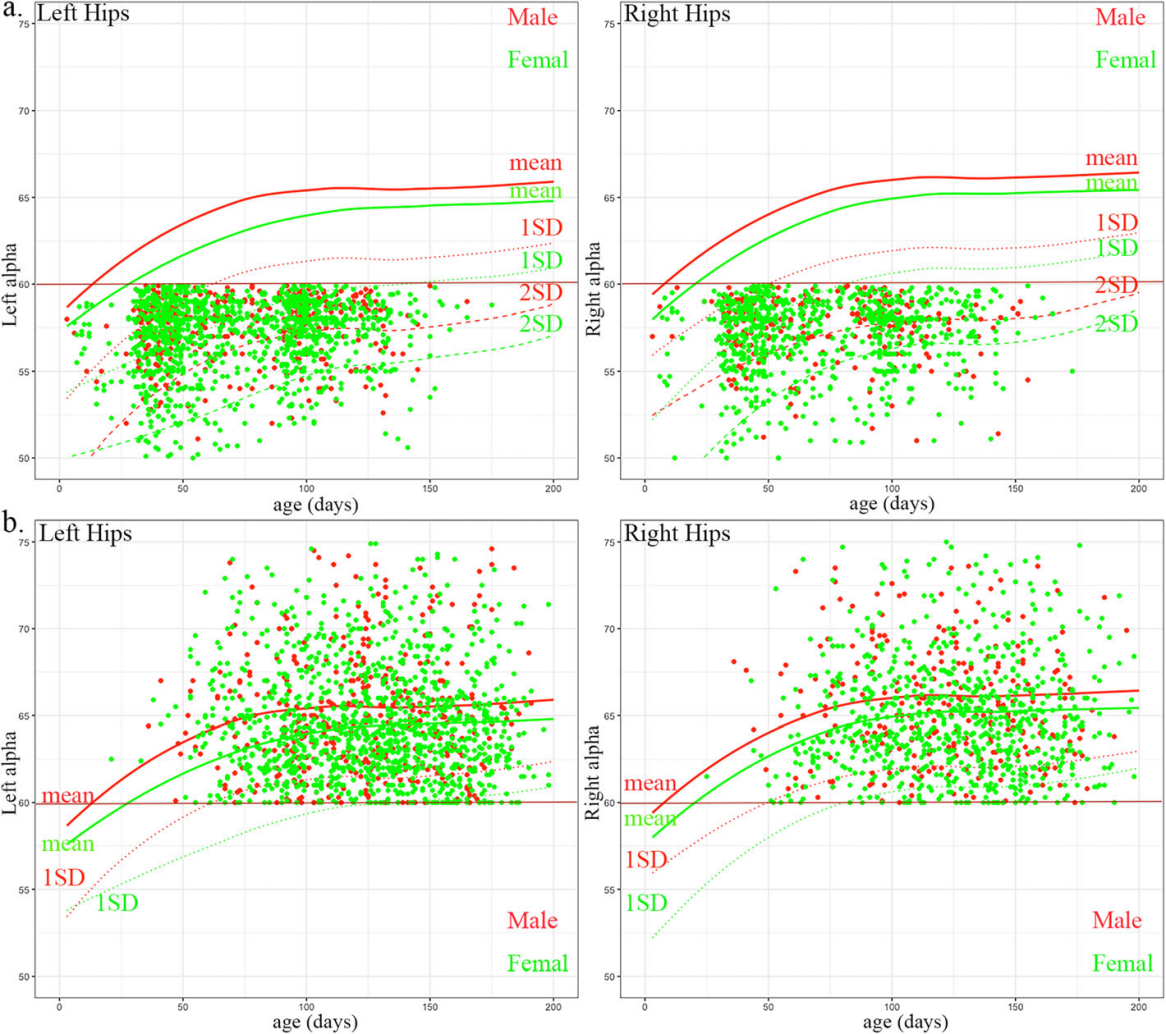
The α angles and ages of the false-positive population. **a** The first examination with positive results. **b** The later examination with negative results. The mean value of Z-score model is shown in solid line. One SD (standard deviation) and 2 SD value of Z-score are shown in dotted and dashed line. The red and green colors represent male and female infants respectively

In the 4229 infants with multiple ultrasonographic hip examinations (a visualization of the first and last examinations is shown in Supplemental Fig. S2 (see Additional file 1)), 1709 infants had negative results of both hips at the first examination and 2520 infants had positive results including 1985 left hips and 1280 right hips (details shown in Supplemental Table S2 (see Additional file 1)). In the positive population, 2024 infants had recovered at the follow-up ultrasonographic examination, including 1630 left hips and 1079 right hips (details shown in Fig. 4, the recovery time of different Graf types is shown in Supplemental Table S3 (see Additional file 1)). Based on this, the false-positive rate of classic Graf classification is 82.12% for left hips and 84.30% for right hips at the first examination. As the two dashed lines that represent the Z = 2 and Z = 1 were plotted in Fig. 4, many of these false-positive hips above these Z-score threshold lines.

**Fig. 5:**
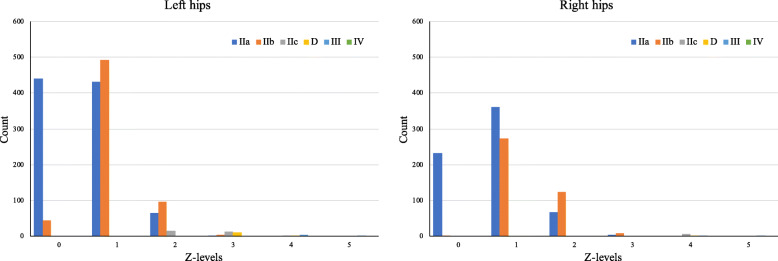
The distribution of false-positive Graf types in Z-levels

The Z-levels of the false-positive results are shown in Fig. 5. If using the Z-scores≥2 as a threshold, the false-positive results can be reduced 86.56% for the left hip and 80.44% for the right hip. Even when using a more sensitive threshold (Z ≥ 1), the false-positive results can be reduced 29.82% for the left hip and 21.78% for the right hip. Both of them could significantly reduce the false-positive rate of the first examination.

In the further evaluation based on follow-up X-ray examination, there are 1803 patients were reported as “no abnormality is revealed” in their X-ray examination in total 5284 patients with follow-up X-ray examinations. Based on their ultrasonographic examinations, there are 276 (15.3% false-positive rate) patients with false-positive in the first US examination. When using the Z-score ≥ 2 as the threshold, the false-positive cases in US examinations were only 58 (3.2% false-positive rate). That is to say total 218 (78.99% of all false-positive cases) false-positive cases will be avoided. However, if using the Z-score ≥ 1 as the threshold, the false-positive cases were 396 (21.96% false-positive rate) and it introduced more false-positive cases.

## Discussion

The three static thresholds (60, 50, 43) of the α angle used in the classic Graf classification were also generated from population data without differentiating race, age, sex, and hip side. Therefore, such fixed and static thresholds lack targeted approaches for specific races, sexes, left and right hips, and age. The introduction of extra Graf types (IIa+,IIa-) did not fundamentally solve this issue. The Z-score models generated in this study have confirmed there are obvious differences among infants with different sexes, ages, and hip sides. There are also racial or ethnic differences based on some studies. The debate over DDH US screening in different countries can be partially explained by these race differences. Thus, why do we still use a fixed threshold in DDH screening for both boys and girls and the left and right hip at all ages in different countries? The Graf method has provided a very standardized protocol to examine and measure the possibility of DDH. Adapting dynamic normal reference values adjusted for side, age, sex, and ethnicity will improve the DDH screening methodology in theory.

In this study, a Z-scores model was established based on a real-world population and it demonstrated its power to control the serious false-positive rate issue using the classic Graf method in DDH screening. Using the widely accepted Z ≥ 2 threshold, the enhanced Graf method can dramatically reduce the false positive rate based on the evaluation. Different countries that concern about the cost and the possibility of overdiagnosis and overtreatment can adjust the Z-score thresholds based on their epidemiology data and healthcare policies. The Z-score thresholds themselves will show what percentage of the population deviates from the specific mean will be screened. As the incidence of DDH in girls was about 5 ~ 9 times higher than it in boys, we also suggest using different Z-score thresholds for boys and girls.

The major challenge of this study is that there is no gold standard for DDH examinations. Not only the early US examination, the radiographs also faced with the challenge of poor concordance between observers and ratings [32]. Some of the infants with positive results would be non-invasive treated to different degrees and these treatments have been approved effective especially for infants within 4–5 months of life [33], such that false-positive rates may be overestimated in this study. Another concern is strict control of the false-positive rate will bring more false-negative cases and will reduce the significance of screening. We did not evaluate the false-negative (missed diagnoses) when using Z-scores in this study for several reasons. First, Z-scores indicate how many standard deviations away from the mean value are. The Z value itself can explain the severe degree and thus severe cases will not be missed. Second, we found most of the α angles grew over time (as shown in Supplemental Fig. S3 (see Additional file 1)). In children who remained positive at the last examination (as shown in Supplemental Table S4 (see Additional file 1)), their α angle may still grow to the normal range. As there are still many borderline values (around 60), we believe some positive results will become negative in later examinations. We also noticed some DDH cases were confirmed in follow-up X-ray examination with very good α and β angles in their early ultrasonographic examination, these cases will be missed no matter how the threshold was defined. Furthermore, the local optimized Z-score based threshold can let the DDH screening program customize their target population for DDH to balance the costs and efficiency.

Another limitation of this study should be noted. As the Z-score model in this study was derived from a population of selected infants, there will be some bias for both the mean and the SD. Considering its relatively large data size of this study and the prevalence of DDH, this bias is acceptable for this demonstration study. However, the reliability of reference data is crucial because important clinical decisions may be based on the interpretation of these measurements. In 2017, the North American Pediatric Heart Network reported Z scores of 2-dimensional echocardiographic measurements derived from over 3000 subjects [34]. In DDH screening, we still lack such a well-controlled population-based Z-scores database to support the Graf method. We hope this study can promote relevant organizations to establish a more accurate and specific DDH screening reference system. Based on a Z-score model generated from a well-controlled population, the threshold can be defined based on the incidence of the diseases. For example, the incidence is 1 in 1000 births in a country, the idea Z-score threshold will be 3. If the incidence is 1 in 100 births in another country, the idea Z-score threshold will be 2.3. These Z-score based thresholds provide a more meaningful way for the policymaker to define the threshold of the screening program.

## Conclusions

The Graf method has been widely used for DDH screening, but there are also concerns about its high false-positive rate in early screening in many countries. In this study, an age, sex, and side specific Z-scores model that was derived from more than 30,000 Chinese children was created and demonstrated an ability to control the false-positive rate of early DDH screening. Introducing Z-scores to build population-specific DDH screening will help reduce the concerns about the cost of the high false-positive rate and promote the popularity of DDH screening programs in additional regions and countries.

## Supplementary Information

**Additional file 1: Figure S1**. The difference of left and right hip. The angle α of right hip is larger than that of left hip in both male and female of all age. **Figure S2**. Visualization of the first and last examination of 4229 infants with follow-up ultrasonographic examinations. A. points were colored in Graf types. B. points were colored in Z-levels. **Figure S3**. The change of angle α in multiple examinations. The x axis represents the age in weeks of the first US examination. The y axis represents the change of angle α value in 30 days at different first examination age. The false positive patients were shown in solid line; The true positive (positive at both first examination and follow-up examination) patients were shown in dash line; All patients were shown in dotted line. **Figure S4**. The difference of the “true positive” and “false positive” population. The upper section shows the “true positive” population at two time points (left and right hip respectively) and the lower section shows the “false positive” population at two time points (left and right hip respectively). The false positive cases concentrated at the early examination and with more border line values. **Table S1** Graf hip classification. **Table S2.** The Graf types of the first-time results in population with follow up US examinations. **Table S3** The Graf types and recover time of the false positive hip. **Table S4** The Graf types of the 551 positive patients at first-time and last-time examination.

## Data Availability

All data generated or analyzed during this study are included in this published article and its supplementary files and online tools (http://hdb.nbscn.org/ddh).

## Abbreviations

DDH: Developmental Dysplasia of the Hip
US: Ultrasonography
SD: Standard deviation
