# Supplementary material for: Using Z-score to optimize population-specific DDH screening: a retrospective study in Hangzhou, China
Source: BMC Musculoskelet Disord. 2021 Apr 12;22:344. doi: 10.1186/s12891-021-04216-6 (PMC8042719; doi:10.1186/s12891-021-04216-6)
Supplement: Supplementary file 1 — Additional file 1: Figure S1. The difference of left and right hip. The angle α of right hip is larger than that of left hip in both male and female of all age. Figure S2. Visualization of the first and last examination of 4229 infants with follow-up ultrasonographic examinations. A. points were colored in Graf types. B. points were colored in Z-levels. Figure S3. The change of angle α in multiple examinations. The x axis represents the age in weeks of the first US examination. The y axis represents the change of angle α value in 30 days at different first examination age. The false positive patients were shown in solid line; The true positive (positive at both first examination and follow-up examination) patients were shown in dash line; All patients were shown in dotted line. Figure S4. The difference of the “true positive” and “false positive” population. The upper section shows the “true positive” population at two time points (left and right hip respectively) and the lower section shows the “false positive” population at two time points (left and right hip respectively). The false positive cases concentrated at the early examination and with more border line values. Table S1 Graf hip classification. Table S2. The Graf types of the first-time results in population with follow up US examinations. Table S3 The Graf types and recover time of the false positive hip. Table S4 The Graf types of the 551 positive patients at first-time and last-time examination. [file 12891_2021_4216_MOESM1_ESM.pdf]

Using Z-score to optimize population-specific DDH screening: a retrospective study in Hangzhou, China

Haomin Li[1*], Liqi Shu[2], Jin Yu[1], Zeng Xian[3], Huilong Duan[3], Qiang Shu[1], Jingjing Ye[1*]

1. The Children's Hospital, Zhejiang University School of Medicine, National Clinical Research Center for Child Health, Hangzhou, China;

2. Rhode Island Hospital, Brown University, United States;

3. The College of Biomedical Engineering and Instrument Science, Zhejiang University, Hangzhou, China;

Supplemental Material

This supplemental material included 4 supplemental figures and 4 supplemental tables:

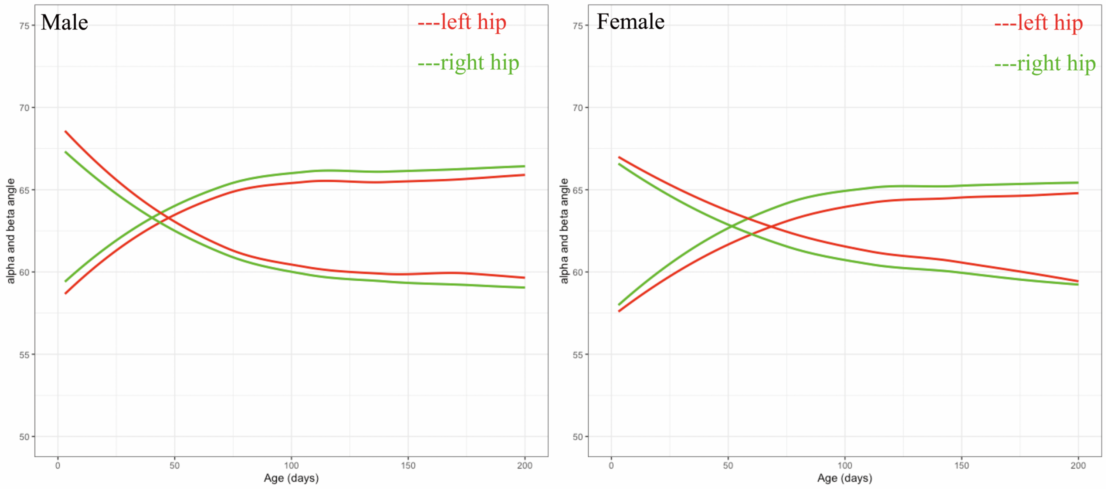

Figure S1 The difference of left and right hip. The angle α of right hip is larger than that of left hip in both male and female of all age.

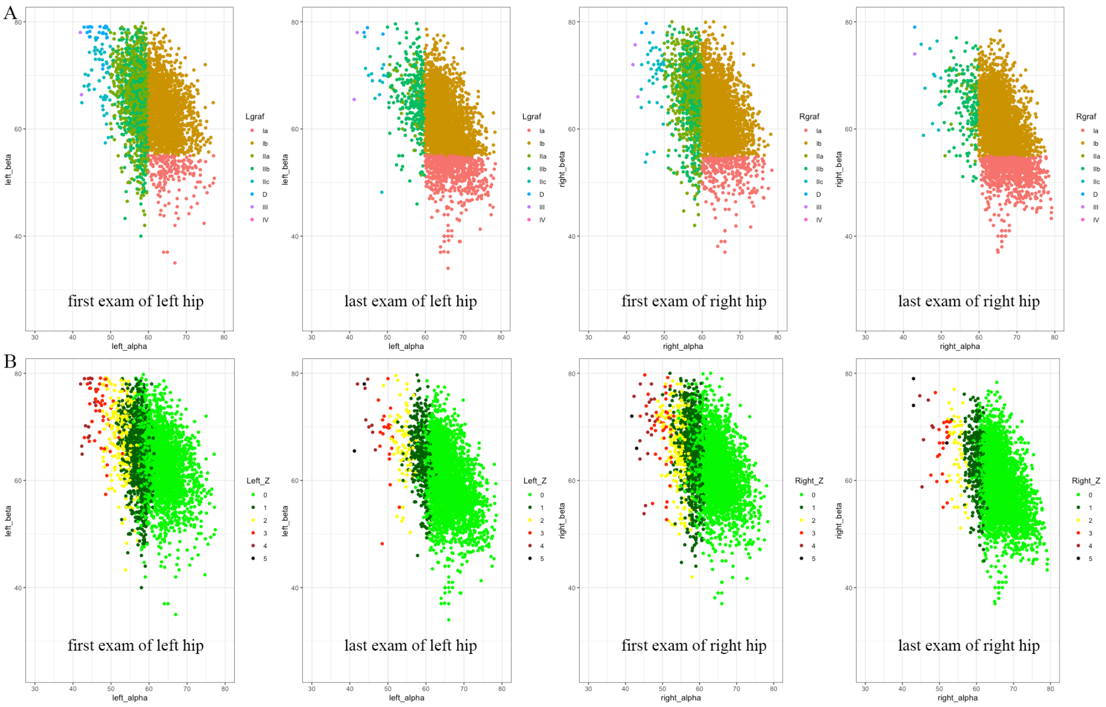

Figure S2. Visualization of the first and last examination of 4229 infants with follow-up ultrasonographic examinations. A. points were colored in Graf types. B. points were colored in Z-levels.

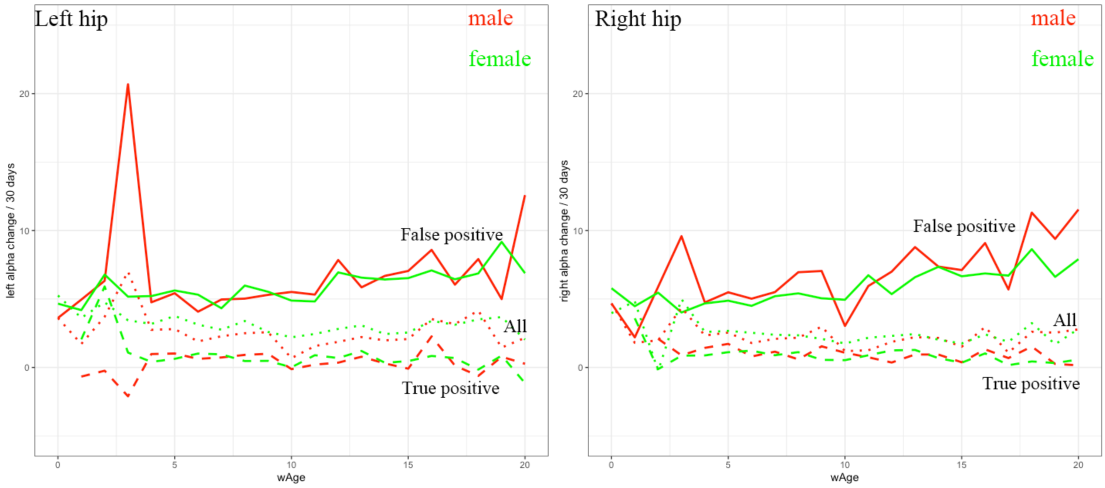

Figure S3. The change of angle α in multiple examinations. The x axis represents the age in weeks of the first US examination. The y axis represents the change of angle α value in 30 days at different first examination age. The false positive patients were shown in solid line; The true positive (positive at both first examination and follow-up examination) patients were shown in dash line; All patients were shown in dotted line.

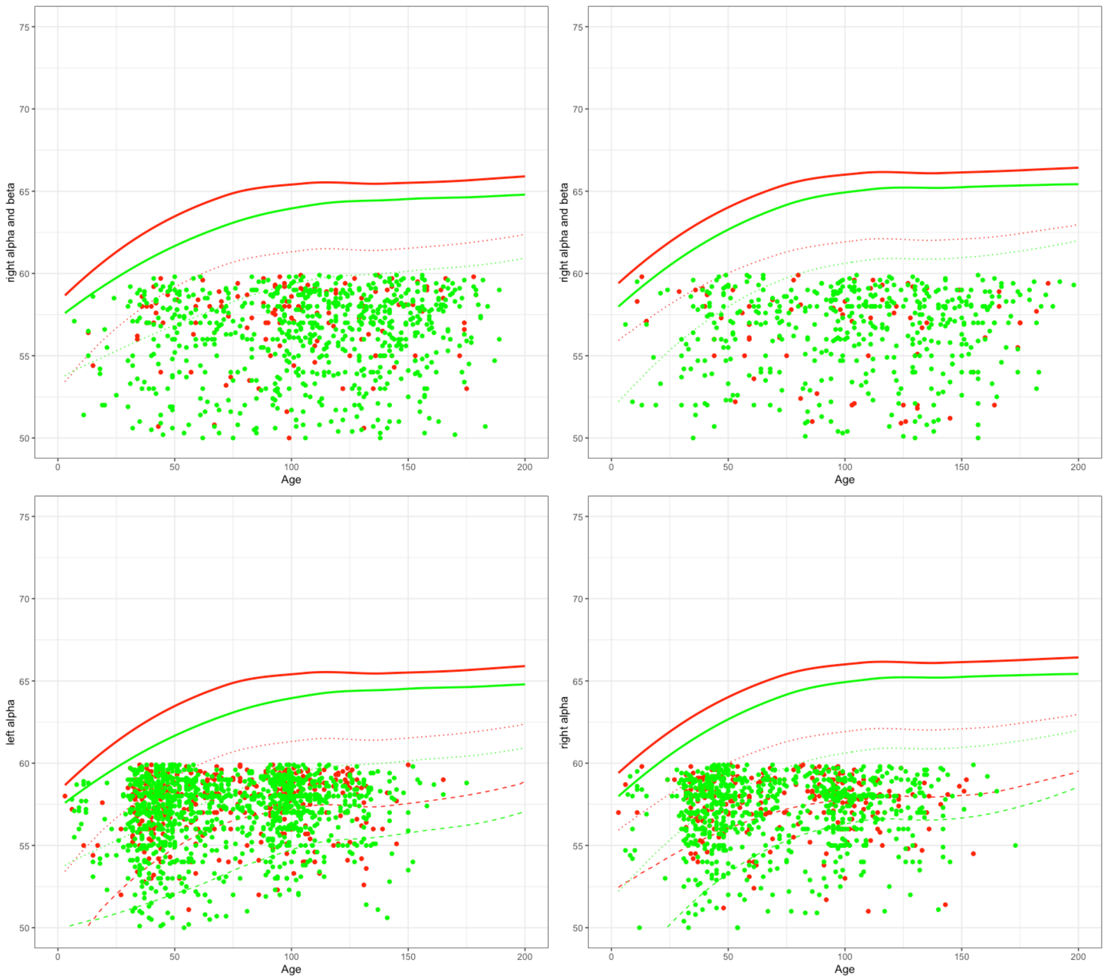

Figure S4. The difference of the “true positive” and “false positive” population. The upper section shows the “true positive” population at two time points (left and right hip respectively) and the lower section shows the “false positive” population at two time points (left and right hip respectively). The false positive cases concentrated at the early examination and with more border line values.

Table S1 Graf hip classification

Hip Type	*α angle*	*β angle*	*Age*	*Description*
**Ia**	≥60°	≤ 55°	All	Mature
**Ib**	≥60°	> 55°	All	Mature
**IIa**	50°~59°		≤3 months	Physiologically immature
**IIb**	50°~59°		>3 months	Delay of ossification
**IIc**	43°~49°	<77°	All	Critical hip
**D**	43°~49°	>77°	All	Decentering hip
**III**	<43°		All	dislocated hip
**IV**	<43		All	dislocated hip

Supplemental Table S2 The Graf types of the first-time results in population with follow up US examinations

		Left Hips				Right Hips			
		Case #	Age (days)	sex		Case #	Age (days)	sex	
				Male	Female			Male	Female
**Total**		4229	80.61409 (±75.00913)	1379	2850	4229	80.61409 (±75.00913)	1379	2850
**Graf**	Ia	209 (4.94 %)	90.66 (±51.58)	96	113	297 (7.02 %)	95.25 (±89.56)	115	182
	Ib	2035 (48.12%)	81.95 (±52.98)	806	1229	2652 (62.71%)	80.23 (±47.96)	967	1685
	IIa	1056 (24.97%)	55.77 (±114.00)	261	795	723 (17.10%)	56.76 (±137.76)	169	554
	IIb	791 (18.70%)	109.31 (±54.48)	206	585	489 (11.56%)	110.26 (±35.87)	119	370
	IIc	59 (1.40%)	72.64 (±31.26)	3	56	37 (0.87%)	74.19 (±28.04)	4	33
	D	39 (0.92%)	71.36 (±32.94)	1	38	11 (0.26%)	56.36 (±34.85)	2	9
	III	34 (0.80%)	67.82 (±27.71)	5	29	14 (0.33%)	65.05 (±33.71)	3	11
	IV	6 (0.14%)	79.33 (±35.85)	1	5	6 (0.14%)	91.57 (±35.75)	0	6

Supplemental Table S3 The Graf types and recover time of the false positive hip

	Left hip		Right hip	
	Case	Recovery time (days)	Case	Recovery time (days)
Total	1630	42.55(±23.22)	1079	39.40 （±21.69）
IIa	938 (57.55%)	47.47(±24.69)	656(60.80%)	43.67(±22.95)
IIb	638(39.14%)	33.91(±17.00)	404(37.44%)	31.95(±16.91)
IIc	28(1.72%)	61.43(±25.23)	13(1.20%)	46.15(±20.28)
D	17(1.04%)	52.53(±23.87)	3(0.28%)	39.67(±14.57)
III	9(0.55%)	65.78(±34.17)	3(0.28%)	78.67(±31.37)
IV	0(0.00%)		0(0.00%)	

Supplemental Table S4 The Graf types of the 551 positive patients at first-time and last-time examination

		T2 (last-time result)		T1 (first-time result)	
		left	right	Left	right
**Graf**	Ia	16 (2.90 %)	41(7.44 %)	15(2.72%)	30(5.44%)
	Ib	125(21.96%)	262(47.55%)	115(20.87%)	229(41.56%)
	IIa	59(10.71%)	43(7.80%)	158(28.68%)	125(22.69%)
	IIb	313(60.07%)	177(32.12%)	177(32.12%)	117(21.23%)
	IIc	16(2.90%)	13(2.36%)	33(5.99%)	25(4.54%)
	D	5(0.91%)	1(0.18%)	22(3.99%)	8(1.45%)
	III	9(1.63 %)	6(1.09%)	25(4.54%)	11(2.00%)
	IV	8(1.45 %)	8(0.14%)	6(1.09%)	6(1.09%)
